# Effectiveness of Message Frame-Tailoring in a Web-Based Smoking Cessation Program: Randomized Controlled Trial

**DOI:** 10.2196/17251

**Published:** 2020-04-03

**Authors:** Maria Altendorf, Ciska Hoving, Julia CM Van Weert, Eline Suzanne Smit

**Affiliations:** 1 Department of Communication Science Amsterdam School of Communication Research University of Amsterdam Amsterdam Netherlands; 2 Department of Health Promotion Care and Public Health Research Institute Maastricht University Maastricht Netherlands

**Keywords:** online computer tailoring, smoking cessation, message frame tailoring, content tailoring, need for autonomy, randomized controlled trial

## Abstract

**Background:**

The content of online computer-tailored interventions is often determined to match an individual’s characteristics, beliefs, and behavioral factors. These content-tailored interventions lead to better message processing and a higher likelihood of behavior change such as smoking cessation. However, a meta-analysis of online computer-tailored interventions showed that effect sizes, albeit positive, remain small, suggesting room for improvement. A promising strategy to enhance the effectiveness of online computer-tailored interventions is to tailor the message frame (ie, how a message is communicated) based on the preferred communication style of the user in addition to content-tailoring. One factor that determines an individual’s communication style preference is the need for autonomy; some individuals prefer an autonomy-supportive communication style (offering choice and use of suggestive language), whereas others might prefer a directive communication style, which is replete with imperatives and does not provide choice. Tailoring how messages are presented (eg, based on the need for autonomy) is called message frame-tailoring.

**Objective:**

The aim of the present study was to test the effectiveness of message frame-tailoring based on the need for autonomy, in isolation and in combination with content-tailoring, within the context of an online computer-tailored smoking cessation intervention. The primary outcome measure was the 7-day point-prevalence of smoking abstinence. Secondary outcomes were perceived message relevance, self-determined motivation to quit smoking, and sociocognitive beliefs.

**Methods:**

A randomized controlled trial with a 2 (message frame-tailoring vs no message frame-tailoring) by 2 (content-tailoring vs no content-tailoring) design was conducted among adult smokers intending to quit smoking (N=273).

**Results:**

Structural equation modeling revealed that the content-tailored condition increased smoking abstinence rates 1 month after the start of the intervention (beta=.57, *P*=.02). However, neither message frame-tailoring nor its interaction with content-tailoring significantly predicted smoking abstinence. In our model, message frame-tailoring, content-tailoring, as well as their interaction significantly predicted perceived relevance of the smoking cessation messages, which consequently predicted self-determined motivation. In turn, self-determined motivation positively affected attitudes and self-efficacy for smoking cessation, but only self-efficacy consequently predicted smoking abstinence. Participants in the control condition perceived the highest level of message relevance (mean 4.78, SD 1.27). However, messages that were frame-tailored for individuals with a high need for autonomy in combination with content-tailored messages led to significantly higher levels of perceived message relevance (mean 4.83, SD 1.03) compared to those receiving content-tailored messages only (mean 4.24, SD 1.05, *P*=.003).

**Conclusions:**

Message frame-tailoring based on the need for autonomy seems to be an effective addition to conventional content-tailoring techniques in online smoking cessation interventions for people with a high need for autonomy; however, this is not effective in its current form for people with a low need for autonomy.

**Trial Registration:**

Dutch Trial Register (NL6512/NRT-6700); https://www.trialregister.nl/trial/6512

## Introduction

Smoking tobacco is the single most preventable cause of noncommunicable diseases such as cancer [1]. Behavioral support through online computer content-tailored (CCT) smoking cessation interventions can be effective in improving quit rates among smokers, substantially exceeding the success rates of more static interventions such as generic online smoking cessation information [2]. Online CCT smoking cessation interventions aim to provide smokers with individualized cessation information, which is assessment-based (eg, a computerized survey assesses participants’ current behavioral beliefs, characteristics, and other attributes) and automatically created by computer software [3-6]. In content-tailored messages, an individual’s responses are automatically matched with the relevant message content only. Previous studies have shown that content-tailored messages increase the perceived message relevance and enhance desired behavior [4,6-10]. Although online CCT smoking cessation interventions lead to better message processing and a higher likelihood of performance of advocated behaviors [5,8,11], effect sizes tend to remain small [2]. To enhance the effectiveness of online CCT health interventions, it is suggested to also use message frame-tailoring in which the message frame is matched (ie, tailor *how* a message is presented or formulated) based on a person’s preferred communication style in addition to message content-tailoring [10-12]. However, no smoking cessation interventions that incorporate both content-tailoring and frame-tailoring have been rigorously tested to date.

A promising factor for message frame-tailoring is people’s need for autonomy (NFA), which determines one’s preference for an autonomy-supportive or more directive communication style, as shown in several studies conducted in face-to-face and other offline health settings in the fields of cancer prevention and healthy nutrition [11,13,14]. In self-determination theory [15], it is theorized that the satisfaction of a person’s NFA is essential for the development of self-determined motivation, well-being, and behavioral engagement [16-18]. In turn, motivations to change are more likely to be translated into actions via sociocognitive beliefs (ie, attitudes, subjective norms, and self-efficacy perceptions) when this motivation is self-determined [19]. People with a higher NFA prefer to choose their own way of how to obtain a goal such as to quit smoking, whereas those with a lower NFA instead prefer to be told through clearcut expert advice how best to reach their goal [11,13,14,19,20]. To illustrate this difference, two studies on the effects of printed health communication showed that people’s preference for a certain communication style moderated the intervention impact [11,13]. That is, people who received messages that were frame-tailored according to their communication style preference (eg, with a high NFA) and were presented with messages in an autonomy-supportive message style using suggestive language (eg, words such as “may” or “could”) more often performed the desired behavior than those who received no frame-tailored messages or messages in a controlling message style (eg, messages in directive wording such as “must” or “should”).

However, to the best of our knowledge, there has been no study investigating whether message frame-tailoring based on the NFA enhances the effectiveness of a content-tailored smoking cessation intervention in an online context. Therefore, the aim of the present study was to test the effectiveness of message frame-tailoring based on the NFA, in isolation and in combination with content-tailoring, within the context of an online CCT smoking cessation intervention. The online environment is specifically promising to enhance intervention effectiveness, as it has a great reach and is thus an “easy to access” medium compared to tailored print health information [21].

**Figure 1:**
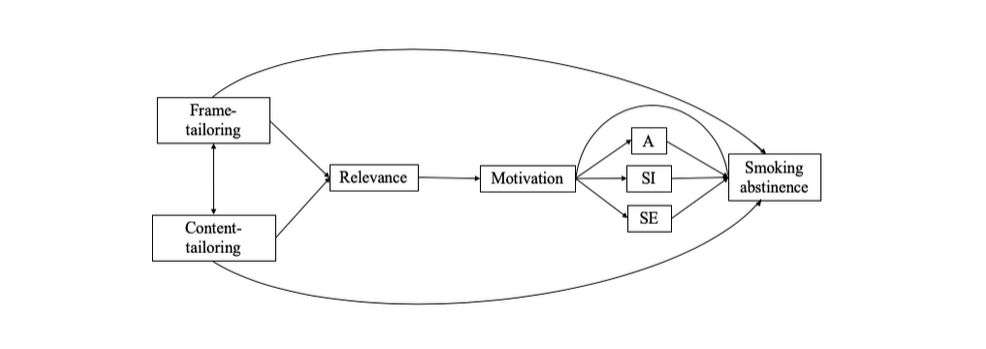
Conceptual model. Smoking abstinence was measured as the 7-day point prevalence of absence of smoking. A, attitudes; SE, self-efficacy; SI, social influence.

Specifically, we set out to test the following three main hypotheses: (H1a) frame-tailoring based on people’s NFA will lead to higher smoking abstinence rates than no frame-tailoring, (H1b) content-tailoring will lead to higher abstinence rates than no content-tailoring, and (H1c) the combination of message frame-tailoring and content-tailoring will lead to the overall highest abstinence rates. In addition, we hypothesized that the above-described effects of message frame-tailoring, content-tailoring, and their combination are mediated by perceived relevance of the message (H2a), self-determined motivation (H2b), and sociocognitive beliefs (H2c). Figure 1 depicts the full conceptual model.

## Methods

### Study Design and Procedure

To test the hypotheses, we relied on data collected within a 6-month randomized controlled trial (RCT) using a 2 (frame-tailoring vs no frame-tailoring) by 2 (content-tailoring vs no content-tailoring) between-subjects design. We here present the data of T0 (baseline measurement), Tl (immediate postintervention follow-up), and T2 (1-month postintervention follow-up) measurements in the context of the Web-based CCT smoking cessation program Personal Advice in Stopping smoking (PAS). PAS was exclusively accessible via the project website [22] and was suitable for computers, laptops, as well as for mobile phones and tablets. Prior to study enrolment, smokers in the Dutch general public were targeted through social media (eg, Facebook, Twitter, LinkedIn), Google advertisements, and Dutch (online) newspapers and radio. Once smokers were willing to participate, they were provided with study information and could give their online informed consent, after which they could create their own username and password. Subsequently (T0), participants were automatically assigned to one of the four conditions through computer randomization and asked to complete the baseline questionnaire (T0), invited to use the intervention, and asked to complete the immediate postintervention evaluation (T1). One month later, they were prompted via email to fill out a brief follow-up questionnaire (T2). Participants received a 10 Euro voucher for their total 45-minute participation when completing the last and third follow-up questionnaire after 6 months.

The study was approved by the Institutional Review Board of the Amsterdam School for Communication Research, University of Amsterdam (2017-PC-7599), and is registered with the Dutch Trial Register (NL6512/NRT-6700).

### Participants

**Figure 2:**
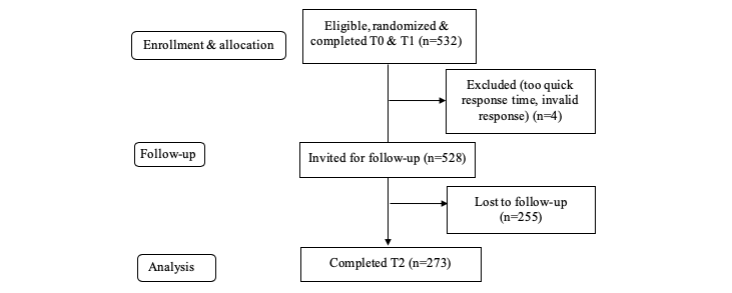
Flow chart of participants. T0, enrollment, allocation, and baseline measurements; T1, measurement of perceived relevance; T2, measurement of motivation, attitude toward smoking cessation, social influence, self-efficacy, smoking abstinence.

At baseline, 534 participants were recruited from mid-December 2018 to March 2019, 273 (51.1%) of whom could be followed-up after 1 month, including 85 (31.1%) in the frame-tailored and content-tailored group, 58 (21.2%) in the frame-tailored and no-content-tailored group, 55 (20.1%) in the control group, and 75 (27.5%) in the no frame-tailored and content-tailored group. The participant flow throughout the study is shown in Figure 2. Inclusion criteria for participants were: 18 years or older, intending to quit smoking within the upcoming 6 months, providing online informed consent, and not having smoked during the last 7 days. An a priori power analysis using G*Power software [23] estimated that a sample size of a minimum of 198 participants should be sufficient to detect small effects and interaction effects (power=.80, odds ratio=1.68, R^2^ content-tailoring=.03) based on an earlier study [24].

### Experimental Conditions

#### Content-Tailoring

In the content-tailored condition*,* participants received smoking cessation advice adapted according to their answers in the questionnaire, which was grounded in the I-Change Model [25]. Questions concerned participants’ smoking behavior, attitude, self-efficacy, social influence, action and coping planning, as well as their intention to quit smoking or to remain a nonsmoker [26,27].

#### Frame-Tailoring

Message frame-tailoring was based on an assessment of the participants’ individual NFA. Participants with a high NFA received autonomy-supportive message frames that encouraged people to accept responsibility for their own behavior by taking the message recipient’s perspective into account through reflective feedback, using language that minimized pressure on the reader, and providing choice (eg, by choosing whether or not to receive additional information on smoking cessation or by choosing whether or not to decide on a quit smoking date) [11,13,28,29]. Participants with a low NFA received controlling message frames that consisted of directive and forceful sentences with imperatives and commands. In addition, authoritative statements such as “experts say” were included and positive filling terms (eg, “luckily”, “good”) were avoided. In this case, the participants were not provided with choice, but rather received all smoking cessation information and a quit date within the next 2 weeks.

As in the frame-tailoring–only condition, in which we tailored both the content and message frames, the style or tone was adjusted based on the NFA throughout all intervention messages.

#### Control

In the control condition, participants received generic smoking cessation advice, which was neither tailored to their preassessed answers nor to their NFA. A smoking cessation advice example used for each of the conditions is shown in Multimedia Appendix 1.

### Pilot Testing

Previous to this study, we conducted an extensive usability test of PAS among smoking cessation experts (N=5) and smokers from different sociodemographic backgrounds (N=7) (personal communication with van Strien-Knippenberg, Faculty of Social and Behavioral Sciences, University of Amsterdam). The questionnaire and stimulus materials were pilot-tested and used in previous online experiments (details can be obtained from the corresponding author MA on request and in our previous study [24]).

### Measures

#### Overall Measures and Evaluation Timeline

At baseline (T0), we measured demographic variables along with the frame-tailoring and content-tailoring variables (ie, NFA and I-Change Model variables). Immediately postintervention (T1), the manipulation assessment and participants’ perceived relevance were measured. At 1-month follow-up (T2), self-determined motivation, sociocognitive beliefs, and smoking abstinence were assessed. All items were measured on a 7-point scale ranging from 1 (strongly disagree) to 7 (strongly agree), unless indicated otherwise. Full descriptions of the scales, including item wording, are listed in Multimedia Appendix 2.

#### Demographics

Age, gender, living arrangement, educational level, presence of respiratory or cardiovascular diseases and (in the case of female gender) pregnancy, and smoking-related behaviors (eg, cigarettes smoked per day) were assessed.

#### Dependent Variable

We measured the 7-day point prevalence abstinence from smoking (smoking abstinence) by asking participants whether they had smoked in the last 7 days (yes=0, no=1).

#### Mediators

The perceived relevance of the smoking cessation message was assessed using 3 items described by Zhao and Peterson [9]. This scale was proven to be reliable, in which higher scores signified higher perceived relevance (Cronbach alpha=.87, mean 4.44, SD 0.08). Self-determined motivation to quit smoking was measured using the 6-item Treatment Self-Regulation Questionnaire (TSRQ) [30], which also showed good reliability (Cronbach alpha=.92, mean 5.34, SD 0.21). Higher scores on the response scale denoted higher levels of self-determined motivation to quit.

Attitudes toward smoking cessation, social influence beliefs, self-efficacy, and intention to quit smoking were assessed based on the I-Change Model [25]. Twelve items were used to measure attitude toward smoking cessation, which were answered on a 5-point Likert scale (1=completely disagree, 5=completely agree). Higher scores indicated higher perceptions of the pros or cons of smoking cessation, respectively. Two subscales were formed with each of the 6 items assessing the perceived pros and cons of smoking cessation, respectively. Both subscales appeared to have good reliability (Cronbach alpha_pros_=.79, mean 3.55, SD 0.62; Cronbach alpha_cons_=.76, mean 2.36, SD 0.51).

Social influence was measured using the concepts of social support (3 items) and social norms (3 items). Answers were given within 6 response categories. The subscales for social support and social norms had poor reliability (Cronbach alpha_social support_=.58; Cronbach alpha_social norms_=.48) and therefore neither of these scales could be used.

Self-efficacy was measured by 9 items, which were answered on a 5-point Likert scale (1=strongly disagree, 5=strongly agree). The scale was found to be reliable (Cronbach alpha=.91, mean 3.51, SD 0.29) and higher scores indicated higher perceived self-efficacy for smoking cessation.

#### Tailoring Variables

NFA was assessed with the Health Causality Orientations Scale (HCOS) [14,31]. In the HCOS, participants receive 4 scenarios for changing their health behavior with each of 3 different statements of how they would act in the scenario (eg, methods of quitting smoking). The participants then have to indicate how they would quit smoking by choosing one of the 3 statements. Each statement comprises a motivation orientation (ie, self-determined, controlled orientation toward friends and family, controlled orientation toward experts). Responses were given on a 5-point Likert scale (1=very unlikely, 5=very likely). Four items from the HCOS reflect people’s autonomous orientation, which were used to determine the participant’s NFA; higher mean scores indicated a higher NFA. For tailoring, the cutoff point to determine a high or low NFA was 3.8 on the HCOS, which was based on results from an earlier online experiment (more details can be obtained from the corresponding author MA on request).

### Manipulation Check

To assess the validity of our frame-tailoring approach, we used 4 items that assessed the degree to which participants perceived the tone of the advice as controlling or autonomy-supportive (eg, “The advice was formulated in a pressuring tone”). The validity of our content-tailored manipulation was measured with 3 items asking whether participants felt that the smoking cessation advice was specifically written for them (eg, “In this program, I received advice based on the responses that I gave to the questions”). Responses were given on a 5-point Likert scale (1=strongly disagree, 5=strongly agree).

### Statistical Analysis

Descriptive analyses with SPSS version 25 (SPSS Inc., Chicago, IL, USA) were conducted to determine sample characteristics and to check for differences in background variables and smoking-related behaviors (eg, number of cigarettes smoked on an average day) between conditions. We used two-sided *t* tests, Chi square tests, and analysis of variance (ANOVA) as appropriate. In addition, a nonresponse analysis with two-sided *t* tests and Chi square tests was conducted to determine whether selective dropout had occurred. We compared complete with lost-to-follow-up cases at T2 with regard to the same set of variables. Structural equation modeling (SEM) was conducted in R (R Foundation for Statistical Computing, Vienna, Austria) with the lavaan package version 0.6-3 [32]. Manifest variables were used for data analysis owing to the rather small sample size (N=273) for SEM analysis. Covariances were added among the two subscales measuring attitude toward smoking cessation, as these subscales measured different parts of the same concept. Next, we built a path model with smoking abstinence (measured at T2) as the main outcome. Based on our hypotheses, we added direct paths from the exogenous variables (ie, frame-tailoring, content-tailoring, and their combination) to smoking abstinence. We then added directs paths from the exogenous variables to perceived relevance and to self-determined motivation, along with a direct path from perceived relevance to self-determined motivation. Direct paths were added from self-determined motivation to attitudes and self-efficacy perceptions and to smoking abstinence. In addition, direct paths were added from attitudes and self-efficacy perceptions to smoking abstinence. The significance level was set at 5% and only the direct unstandardized effects are reported.

The data that support the findings of this study are available via Open Science Framework [33].

## Results

### Randomization and Manipulation Check

There were no significant differences between participants in the experimental conditions and control condition with regard to their demographics such as age and educational level, chronic diseases, and smoking behaviors. In terms of the manipulation, as expected, the frame-tailored and content-tailored conditions were significantly more often perceived as such by participants compared to the nonframe-tailored and noncontent-tailored conditions, respectively. Thus, the manipulation succeeded. An overview of all items assessing our manipulations, together with their mean values in each of the experimental conditions, is provided in Multimedia Appendix 3.

### Sample Characteristics and Attrition

Comparisons of the 273 participants who completed the study and the 255 participants who were lost to follow-up after 1 month showed no significant differences in gender, educational level, smoking behaviors, and chronic diseases, but the Chi square test for condition and intervention drop-out was significant (Chi-square_3_=11.15, N=528, *P*=.11): less participants in the message frame-tailoring and content-tailoring condition were lost to follow-up (n=50, 9.5%) compared to participants who received message frame-tailoring without content-tailoring (n=65, 12.3%), generic advice (ie, the control condition; n=71, 13.4%), or no message frame-tailoring but content-tailoring only (n= 69, 13.1%). Participants who dropped out (mean 40.11 years, SD 14.28) were also significantly (*F_1_*=5.89, *P*=.02) younger than those who completed the follow-up measurement (mean 43.05 years, SD 13.52).

Participant age was added as a covariate to our structural model because it was significantly correlated with smoking abstinence and with intervention drop-out. As our model with the covariate was very complex, for clarity purposes, we here only report the results of variables that were of substantial interest based on the theory. Table 1 provides an overview of the sample characteristics of the participants who completed the study and those lost to follow-up. The assumptions of multivariate normality and linearity were met and no multicollinearity existed. We conducted our SEM analysis with the diagonal weighted least squares (DWLS) estimator, which provides robust values from the full weight matrix to compute standard errors. No missing data among endogenous variables were observed.

**Table 1:** Comparison of participants who completed the study with those who dropped out.

Participant characteristics		T0 (N=528)	Completed T2 (N=273)	Dropout at T2 (N=255)
**Demographics**				
	Female, n (%)	187 (35.4)	106 (38.8)	81 (31.8)
	Age (years), mean (SD)	41.63 (13.95)	43.05 (13.526)	40.11 (14.28)^a^
**Educational level, n (%)**				
	High	233 (44.1)	133 (48.7)	100 (39.2)
	Middle	228 (43.2)	111 (40.7)	117 (45.9)
	Low	67 (12.7)	29 (10.6)	38 (14.9)
	Other/missing	0 (0)	0 (0)	0 (0)
**Living arrangement, n (%)**				
	With partner	110 (20.8)	61 (22.3)	49 (19.2)
	With partner and child(ren)	119 (22.5)	61 (22.3)	58 (22.7)
	With child(ren)	55 (10.4)	25 (9.2)	30 (11.8)
	Alone	208 (39.4)	110 (40.3)	98 (38.4)
	Other/missing	36 (6.8)	16 (5.9)	20 (7.8)
**Number of daily smoked, mean (SD)**				
	Cigarettes	11.13 (8.34)	10.6 (7.92)	11.63 (8.75)
	Shags	3.69 (8.26)	3.8 (8.40)	3.75 (8.12)
	Cigars	0.40 (2.68)	0.24 (1.85)	0.57 (3.34)
	Cigarillos	0.18 (1.38)	0.19 (1.34)	0.18 (1.42)
	Pipes	0.13 (1.17)	0.07 (0.78)	0.18 (1.49)
Earlier quit attempts, mean (SD)		5.74(0.58)	5.29 (11.41)	6.23 (15.27)
**Existence of (chronic) disease, n (%)**				
	Heart disease	39 (7.4)	21 (7.7)	18 (7.1)
	COPD^b^	106 (20.1)	58 (21.2)	48 (18.8)
	Diabetes	25 (4.7)	13 (4.8)	12 (4.7)
	Cancer	29 (5.5)	12 (4.4)	17 (6.7)

^a^Significant mean difference from T2: F_1, 526_=5.893, *P*=.02.

^b^COPD: chronic obstructive pulmonary disorder.

We identified outliers among endogenous variables (ie, perceived relevance, self-determined motivation, perceived pros of smoking cessation, self-efficacy), which were checked and considered random, and therefore not removed.

### Model Testing

Our hypothesized path model appeared to have a poor model fit according to conventional goodness-of-fit indices [34]. Based on the modification indices and the residual covariance matrix, we assumed it to be necessary to trim our path model by discarding a variable from the model (ie, the cons of smoking cessation), which subsequently led to good model fit. Table 2 provides an overview of model fit indices for the hypothesized and fitted model.

**Figure 3:**
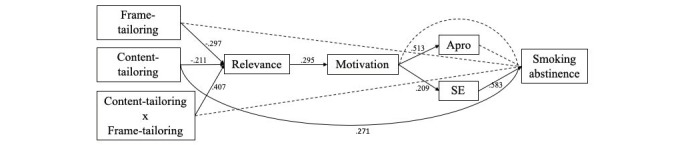
Final model with significant paths only. Results are presented as standardized direct effects. Dotted lines represent nonsignificant paths. Straight lines represent significant paths (*P*&lt;.05). Apro, pros of smoking cessation; SE, self-efficacy.

The results from SEM analysis are depicted in the structural model in Figure 3. For clarity, we present the results only for the significant regression coefficients.

**Table 2:** Fit indices of the path model with smoking abstinence as outcome.

Fit indices	Hypothesized model	*P* value	Final (trimmed) model	*P* value
Chi-square	75.43_18_	<.001	20.249_13_	.09
Comparative fit	.814	N/A^a^	.967	N/A
RMSEA^b^ (90% CI)	0.10 (0.08, 0.13)	N/A	0.04 (0.00- 0.08)	N/A

^a^Not applicable.

^b^RMSEA: root mean square error of approximation.

### Hypothesis Testing

#### Effects of Message Frame-Tailoring and Content-Tailoring on Smoking Abstinence

In contrast to our expectations, neither message frame-tailoring based on a smoker’s NFA nor the combination of message frame-tailoring and content-tailoring significantly affected smoking abstinence. However, as expected, we identified a significant positive effect from content-tailoring on smoking abstinence (beta=.57, *P*=.02). In the frame-tailored and content-tailored condition, 23 (30.3%) smokers refrained from smoking, while 12 (15.8%) smokers in the frame-tailored–only, 11 (14.5%) smokers in the control condition, and 30 (39.5%) smokers in the content-tailored–only condition refrained from smoking. Thus, we could only partly confirm the first hypothesis (H1b).

#### Mediation of Perceived Relevance, Self-Determined Motivation, and Sociocognitive Beliefs About Smoking Cessation

As shown in the model (Figure 3), we identified a significant main effect of content-tailoring and message frame-tailoring based on the users’ NFA as well as their combination on smokers’ perceived relevance of the smoking cessation message, the first mediator in our model. ANOVA showed significant differences between the conditions (*F*_3,269_=4.82, *P*=.003) and Tukey’s post hoc test was used to identify the conditions with significant differences. The control condition (ie, no content- or message frame-tailoring) was perceived as significantly more relevant than message frame-tailoring or content-tailoring alone (mean difference 0.65, SE 0.20 and mean difference 0.54, SE 0.19, respectively) and was similarly relevant as the condition with both content- and message frame-tailoring. Thus, surprisingly, generic smoking cessation advice led to similarly high levels of perceived message relevance as a message that was tailored both in terms of content and message framing, and led to higher perceived relevance than messages tailored in terms of only one of these aspects.

As these findings were against the expected direction, we decided to inspect the data even more closely by comparing participants with a high and low NFA within the message frame-tailored conditions. This comparison showed that smokers with a high NFA generally perceived their message as more relevant compared to participants who had a low NFA when they received a frame-tailored smoking cessation message, both with and without content-tailoring. Moreover, the combination of message frame-tailoring and content-tailoring led to significantly higher perceived relevance than content-tailored messages only, but only for smokers with a high NFA (mean difference 0.59, SE 0.19; *P*=.04). In addition, those with a high NFA in the frame-tailored and content-tailored condition perceived the messages as significantly more relevant compared to those with a low NFA who received frame-tailored but not content-tailored messages. To illustrate these findings, the means per condition for all continuous variables in the SEM are provided in Table 3.

**Table 3:** Mean (SD) values per condition for all endogenous variables (N=273).

Dependent variable	Frame-tailoring and content-tailoring			Frame-tailoring and no content-tailoring			No frame-tailoring and no content-tailoring	No frame-tailoring and content-tailoring	Overall mean	*F*_df_; *P* value^a^
	All participants	High NFA^b^	Low NFA	All participants	High NFA	Low NFA				
										
Relevance	4.59 (1.05)	4.83 (1.03)	4.26 (1.01)	4.13 (0.96)	4.29 (1.07)	3.97 (.83)	4.78 (1.27)	4.24 (1.05)	4.43 (1.10)	4.36_5_; .008^c^, .03^d^, .01^e^
Motivation	5.33 (1.32)	5.57 (1.42)	5.00 (1.10)	5.16 (1.11)	5.36 (1.00)	4.98 (1.19)	5.42 (1.14)	5.42 (1.26)	5.34 (1.22)	1.56_5_; .17
SE^f^	3.52 (0.84)	3.70 (0.82)	3.27 (0.82)	3.47 (0.69)	3.50 (0.56)	3.44 (0.81)	3.48 (0.83)	3.54 (0.96)	3.51 (0.84)	1.12_5_; .35
Apro^g^	3.64 (76)	3.87 (0.74)	3.33 (0.69)	3.43 (0.96)	3.55 (0.84)	3.32 (1.06)	3.55 (0.91)	3.52 (0.86)	3.55 (0.86)	2.26_5_; .05
Acon^h^	2.36 (0.83)	2.21 (0.84)	2.56 (0.80)	2.22 (0.77)	2.13 (0.79)	2.31 (0.76)	2.43 (0.90)	2.39 (0.94)	2.35 (0.86)	1.16_5_; .33

^a^Analysis of variance based on the six groups of subtailoring.

^b^NFA: need for autonomy.

^c^High NFA vs Low NFA frame-tailoring and content-tailoring.

^d^High NFA frame-tailoring and content-tailoring vs no frame-tailoring and content-tailoring.

^e^Low NFA and content-tailoring vs no frame-tailoring and no-content tailoring.

^f^SE: self-efficacy.

^g^Apro: attitudes about pros of smoking cessation.

^h^Acon: attitudes about cons of smoking cessation.

Subsequently, perceived relevance had a positive effect on the self-determined motivation to quit smoking (beta=.32, *P*<.001). Furthermore, although self-determined motivation did not have a direct effect on smoking abstinence, there was a positive effect of self-determined motivation on the perceived pros of smoking cessation (ie, positive attitudes) (beta=.37, *P*<.001) and a positive effect on self-efficacy perceptions (beta=.15, *P*<.001). Moreover, we confirmed a positive effect from self-efficacy perceptions on smoking abstinence (beta=.72, *P*<.001).

In summary, the effect of message frame-tailoring, content-tailoring, as well as their combined effect on smoking abstinence was mediated by perceived relevance, self-determined motivation to quit, and self-efficacy on smoking abstinence. As such, we could confirm the second hypothesis. An overview of the hypothesized direct and indirect effects from our final model is provided in Table 4, and the correlation matrix of standardized effects is provided in Multimedia Appendix 4.

**Table 4:** Standardized indirect and direct effects of the trimmed model^a^.

Independent variable		Perceived relevance	Self-determined motivation	Attitudes	Self-efficacy	Smoking abstinence
**Content-tailoring**						
	Indirect effect	–^b^	–0.062	0.089	–0.001	0.230
	Direct effect	–0.474	–	–	–	0.271
**Frame-tailoring**						
	Indirect effect	–	-0.088	0.064	–0.026	–0.043
	Direct effect	–0.297	–	–	–	0.023
**Perceived relevance**						
	Indirect effect	–	–	0.151	0.062	0.021
	Direct effect	–	0.295	–	–	–
**Self-determined motivation**						
	Indirect effect	–	–	–	–	0.052
	Direct effect	–	–	0.513	0.209	0.072
**Attitudes**						
	Indirect effect	–	–	–	–	–
	Direct effect	–	–	–	–	–0.173
**Self-efficacy**						
	Indirect effect	–	–	–	–	–
	Direct effect	–	–	–	–	0.583
Smoking abstinence						

^a^The model controlled for age, and only the paths to self-determined motivation, social norms, and smoking abstinence were significant.

^b^Not applicable.

## Discussion

### Effect of Tailoring on Smoking Abstinence and Perceived Relevance

The aim of this study was to test the effectiveness of message frame-tailoring based on smokers’ NFA in isolation and in combination with content-tailoring in the context of an online CCT smoking cessation intervention. Our results confirm findings from earlier research on content-tailoring [2,24], as we could identify a positive effect of content-tailoring on 7-day point prevalence abstinence rates 1 month after the start of the intervention. However, in contrast to our expectations, message that were frame-tailored based on the NFA did not lead to higher smoking abstinence rates in isolation and in combination with content-tailoring as compared to the no frame-tailoring condition.

### Mediating Roles of Perceived Relevance, Self-Determined Motivation, and Sociocognitive Beliefs

Overall, our findings were in line with our hypothesis that perceived relevance, self-determined motivation to quit, and sociocognitive beliefs mediate the effects of content-tailoring, message frame-tailoring, and their combination on smoking abstinence. That is, we demonstrated a positive effect of perceived message relevance on the self-determined motivation to quit smoking. Therefore, we could confirm earlier findings concerning elaboration likelihood model research [35] demonstrating that people who perceive their messages as relevant are also more motivated to devote more cognitive effort on processing the messages. Moreover, self-determined motivation positively predicted positive attitudes and self-efficacy beliefs of smoking cessation. Finally, we found a positive effect of self-efficacy on smoking abstinence, which was also observed in a meta-analysis on the integration of self-determination theory and the theory of planned behavior [19].

However, in contrast to our expectations, attitudes toward smoking cessation did not significantly predict smoking abstinence, which is not supported by earlier research [19]. According to the theory of planned behavior [36], positive attitudes lead to an enhanced intention for behavior change, which in turn predicts behavior change. However, previous smoking cessation research showed that self-efficacy perceptions were the main predictors of smoking cessation among smokers intending to quit [37]. A potential explanation for the lack of a significant effect of attitude on smoking abstinence could be that we only used one subscale of attitudes (ie, the pros of smoking cessation). The subscale related to the cons of smoking cessation had to be discarded during SEM analysis owing to the noise it caused in the data, which prevented reaching model fit. This may have led to the attitude variable, as included in our model, not being fully representative of the theoretical construct in its entirety. In addition, we did not assess intention to quit smoking in our study, and therefore cannot state whether attitudes might have indirectly—instead of directly—predicted smoking abstinence, which could have been assumed based on theory and evidence [37,38].

Moreover, we had to discard the social influence scale [39,40] as it had poor reliability (see Multimedia Appendix 2). Thus, we could not test for a possible mediation effect of social influence, which might be an important factor in explaining variance in smoking abstinence rates [41]. Based on comments made by smokers and experts in the pilot testing phase of our intervention, we recommend that efforts be made in future research to improve the comprehensibility, and subsequently the reliability, of the social influence scale by adapting the response categories of the subscales (eg, specification of terms such as “majority of your children” that seemed to be difficult to answer when having two children) and to include the resulting reliable scale in further analyses similar to those presented here.

### Exploring Message Frame-Tailoring on the Need for Autonomy

We found that content-tailoring, message frame-tailoring, and their combination had a significant effect on participants’ perceived message relevance. However, these effects were against expectation as both message frame-tailoring and content-tailoring led to significantly lower perceived message relevance compared to the control condition (ie, generic smoking cessation messages). This finding also conflicts with earlier tailoring-based research [4,8,11], which demonstrated that content-tailored messages led to better message processing, better message recall, and more positive behavioral outcomes via more perceived message relevance. To gain better understanding of this finding, we checked the time the participants took to finish the intervention. Although the control condition messages were similar in length and contained generic smoking cessation information, we wanted to explore whether smokers in the control condition had processed the messages longer and perhaps more thoroughly, resulting in their perceptions of relevance being higher than in the other conditions. However, as participants between conditions did not differ significantly in the time they used to finish the intervention (data not shown), this possibility is unlikely.

An alternative potential explanation comes from exploratory data analyses conducted with the participants’ showing a high and low NFA separated within the message frame-tailored conditions. These analyses showed that participants with a high NFA who received message frame-tailoring (ie, messages in an autonomy-supportive frame) as an addition to content-tailoring did perceive their messages as significantly more relevant compared to participants with a low NFA who received message frame-tailoring and to participants that received messages that were content-tailored only. This finding supports the results of Resnicow et al [11] who showed that participants with a preference for an autonomous form of communication perceived autonomy-supportive messages as more relevant compared to those with a preference for controlling forms of communication. In addition, although not significant, when comparing the means among all other mediators, participants with a high NFA generally had a higher level of self-determined motivation, more positive attitudes about smoking cessation, and higher self-efficacy perceptions compared to those with a lower NFA, regardless of whether or not they had received content-tailored messages.

This pattern raises the question as to whether smokers with a lower NFA might prefer different message frames than those provided in our study (ie, message frames using controlling language and without the provision of choice). It could be that the controlling language that was used might have been too controlling, resulting in message resistance and an insufficient ability to motivate participants with a low NFA to refrain from smoking [42]. Negative message evaluations such as resistance have been shown to lead to less deep message processing [8], resulting in lower message effectiveness. Thus, participants with a low NFA might need differently tailored message frames (eg, clearcut expert advice about smoking cessation using less controlling language such as without imperatives or terms like “must” and “should”). Furthermore, future research would benefit from validating the HCOS scale, which was used to assess the NFA in this study, and further investigate whether the dichotomization of people into high and low NFA based on the 4 items that assess autonomous orientation proofs is a valid categorization approach. Perhaps there is another group (eg, with a moderate NFA) of people or it is better to take into account the HCOS items that assess people’s controlled or impersonal orientations [14]. Such research efforts would help to optimize message frame-tailoring based on the NFA and enable further research into its effectiveness.

Another possibility is that participants with a lower NFA might be less susceptible to autonomy-supportive messages framed in a controlling message style than those with a high NFA owing to, for instance, different message processing needs that potentially correlate with the NFA such as the need for cognition. For example, message frames for low NFA participants might not have sufficiently met their relatively lower autonomy needs or their preferences for (low levels of) information processing. In a similar vein, it could be expected that those with a higher NFA might have better health outcomes than their low NFA counterparts, as individuals with a high NFA might also report higher needs for cognition and thus be more motivated to process health messages and eventually change their health behavior than low NFA individuals [8]. In support of this reasoning, in an exploratory data analysis (data not shown), we found that participants with a high NFA also had higher, but nonsignificant, rates of smoking abstinence than those with a low NFA. Accordingly, it seems to be necessary to study whether the NFA indeed correlates with the need for cognition and how their potential interaction might influence message effectiveness. Moreover, we recommend future research to (qualitatively) investigate how messages should be formulated among those with a lower NFA (and potentially a low need for cognition) to meet their communication preferences and message processing needs so that these messages can lead to optimal health outcomes.

### Study Strengths and Limitations

Findings from this study contribute to a growing understanding of the effects of message frame-tailoring; however, approximately 50% of the participants could not be followed up 1 month after the baseline measurement. Although this is common for RCTs, and in particular for internet-based longitudinal studies [43,44], the high rates of attrition (while also differing between conditions) may have reduced the internal validity of the results presented, consequently introducing potential bias to the estimates of effectiveness. This could result in an overestimation of the effectiveness for several reasons. First, participants who were lost to follow-up were significantly younger than those that completed the intervention. However, this age difference was only 3 years (ie, drop-out age 40 years vs completer age 43 years) and also within a rather homogenous age group. Therefore, realistically, we do not assume major differences among completers and those lost to follow-up. Second, a significantly lower rate of study dropout was observed in the message frame-tailoring and content-tailoring condition, which was expected to impact smoking cessation and underlying mechanisms (eg, attitude change and self-efficacy perceptions) most positively (as compared to other conditions) (see Sample Characteristics and Attrition in the Results section). However, overall, the message frame-tailoring and content-tailoring condition had no significant effect on smoking cessation and underlying mechanisms. Thus, we assume the risk of bias due to study attrition to be small.

To prevent high attrition rates in the first place, we used strategies such as sending several email reminders to participants, and only including participants who were sufficiently motivated to participate, as well as offering shopping vouchers after completion of the intervention and follow-up questionnaires. Moreover, we used a forced data entry option, so that we only had missing values on outcome variables when the entire case was missing and there were no missing values, such as for any of the mediating variables while data on smoking abstinence was present or vice versa. Although we acknowledge that the complete case analysis we conducted might have led to a bias in the results presented, imputing the missing values for the nearly 50% of cases that dropped out of the study would have introduced a high degree of uncertainty that would further reduce the reliability of the presented results. However, a major strength of this study was that we were able to recruit a large sample of eligible smokers, resulting in a sample size that was still sufficient for analysis according to our a priori power analysis.

### Conclusion

This study extends the tailoring literature by providing first evidence for the effects of message frame-tailoring based on the NFA in isolation and in combination with content-tailoring. Based on our findings, we can conclude that message frame-tailoring based on the NFA seems to be an effective addition to conventional content-tailoring techniques in online health interventions for people with a high NFA, but is not effective in its current form for people with a low NFA. To enhance the effectiveness of message frame-tailoring, future research efforts might therefore want to focus on (qualitatively) investigating which type of message frame might be most beneficial for smokers with a low NFA.

## Appendix

Multimedia Appendix 1 Example advice.

Multimedia Appendix 2 Item wording and reliability.

Multimedia Appendix 3 Manipulation check.

Multimedia Appendix 4 Correlation matrix.

Multimedia Appendix 5 CONSORT-eHealth checklist (V 1.6.1).

## Abbreviations

ANOVA: analysis of variance
CCT: computer content tailoring
DWLS: diagonal weighted least squares
HCOS: Health Causality Orientations Scale
NFA: need for autonomy
PAS: Personal Advice in Stopping smoking
RCT: randomized controlled trial
RMSEA: root mean square error of approximation
SEM: structural equation modeling
TSRQ: Treatment Self-Regulation Questionnaire

## References

[1] World Health Organization (WHO) (2014). http://www.euro.who.int/__data/assets/pdf_file/0009/248418/European-Tobacco-Control-Status-Report-2014-Eng.pdf.

[2] Lustria MLA, Noar SM, Cortese J, Van Stee SK, Glueckauf RL, Lee J (2013). A meta-analysis of web-delivered tailored health behavior change interventions. J Health Commun.

[3] de Vries H, Brug J (1999). Computer-tailored interventions motivating people to adopt health promoting behaviours: introduction to a new approach. Patient Educ Couns.

[4] Hawkins RP, Kreuter M, Resnicow K, Fishbein M, Dijkstra A (2008). Understanding tailoring in communicating about health. Health Educ Res.

[5] Kreuter MW, Wray RJ (2003). Tailored and targeted health communication: strategies for enhancing information relevance. Am J Health Behav.

[6] Noar SM, Harrington NG, Aldrich RS (2016). The Role of Message Tailoring in the Development of Persuasive Health Communication Messages. Annal Int Commun Assoc.

[7] Noar SM, Benac CN, Harris MS (2007). Does tailoring matter? Meta-analytic review of tailored print health behavior change interventions. Psychol Bull.

[8] Petty RE, Cacioppo JT (2020). The elaboration likelihood model of persuasion. Communication And Persuasion: Central And Peripheral Routes To Attitude Change (Springer Series In Social Psychology).

[9] Zhao X, Peterson E (2017). Effects of Temporal Framing on Response to Antismoking Messages: The Mediating Role of Perceived Relevance. J Health Commun.

[10] Rimer BK, Kreuter MW (2006). Advancing tailored health communication: A persuasion and message effects perspective. J Commun.

[11] Resnicow K, Davis RE, Zhang G, Konkel J, Strecher VJ, Shaikh AR, Tolsma D, Calvi J, Alexander G, Anderson JP, Wiese C (2008). Tailoring a fruit and vegetable intervention on novel motivational constructs: results of a randomized study. Ann Behav Med.

[12] Smit E, Linn A, vanWeert J (2015). Taking online computer-tailoring forward. Eur Health Psychol.

[13] Resnicow K, Zhou Y, Hawley S, Jimbo M, Ruffin MT, Davis RE, Shires D, Lafata JE (2014). Communication preference moderates the effect of a tailored intervention to increase colorectal cancer screening among African Americans. Patient Educ Couns.

[14] Smit ES, Bol N (2019). From self-reliers to expert-dependents: identifying classes based on health-related need for autonomy and need for external control among mobile users. Media Psychol.

[15] Ryan RM, Deci EL (2000). Self-determination theory and the facilitation of intrinsic motivation, social development, and well-being. Am Psychol.

[16] Ng JYY, Ntoumanis N, Thøgersen-Ntoumani C, Deci EL, Ryan RM, Duda JL, Williams GC (2012). Self-Determination Theory Applied to Health Contexts: A Meta-Analysis. Perspect Psychol Sci.

[17] Williams G, McGregor H, Sharp D, Levesque C, Kouides R, Ryan R, Deci EL (2006). Testing a self-determination theory intervention for motivating tobacco cessation: supporting autonomy and competence in a clinical trial. Health Psychol.

[18] Williams G, Gagné M, Ryan R, Deci E (2002). Facilitating autonomous motivation for smoking cessation. Health Psychol.

[19] Hagger M, Chatzisarantis N (2009). Integrating the theory of planned behaviour and self-determination theory in health behaviour: a meta-analysis. Br J Health Psychol.

[20] Gagné M, Deci E (2005). Self-determination theory and work motivation. J Organiz Behav.

[21] Taylor G, Dalili M, Semwal M, Civljak M, Sheikh A, Car J (2017). Internet-based interventions for smoking cessation. Cochrane Database Syst Rev.

[22] PAS.

[23] Faul F, Erdfelder E, Lang A, Buchner A (2007). G*Power 3: a flexible statistical power analysis program for the social, behavioral, and biomedical sciences. Behav Res Methods.

[24] Smit ES, de Vries H, Hoving C (2012). Effectiveness of a Web-based multiple tailored smoking cessation program: a randomized controlled trial among Dutch adult smokers. J Med Internet Res.

[25] de Vries H (2017). An Integrated Approach for Understanding Health Behavior; The I-Change Model as an Example. Psychol Behav Sci Int J.

[26] Smit E, Candel M, Hoving C, de Vries H (2016). Results of the PAS Study: A Randomized Controlled Trial Evaluating the Effectiveness of a Web-Based Multiple Tailored Smoking Cessation Program Combined With Tailored Counseling by Practice Nurses. Health Commun.

[27] Smit E, de Vries Hein, Hoving C (2010). The PAS study: a randomized controlled trial evaluating the effectiveness of a web-based multiple tailored smoking cessation programme and tailored counselling by practice nurses. Contemp Clin Trials.

[28] Deci EL, Eghrari H, Patrick BC, Leone DR (1994). Facilitating internalization: the self-determination theory perspective. J Pers.

[29] Markland D, Ryan R, Tobin V, Rollnick S (2005). Motivational Interviewing and Self–Determination Theory. J Soc Clin Psychol.

[30] Levesque CS, Williams GC, Elliot D, Pickering MA, Bodenhamer B, Finley PJ (2007). Validating the theoretical structure of the Treatment Self-Regulation Questionnaire (TSRQ) across three different health behaviors. Health Educ Res.

[31] Smit E, Zeidler C, Resnicow K, de Vries H (2019). Identifying the Most Autonomy-Supportive Message Frame in Digital Health Communication: A 2x2 Between-Subjects Experiment. J Med Internet Res.

[32] Rosseel Y (2012). lavaan: An R Package for Structural Equation Modeling. J Stat Soft.

[33] Altendorf M Open Science Framework.

[34] Bryne B (2016). Structural Equation Modeling With AMOS.

[35] Petty RE, Heesacker M, Hughes JN (1997). The elaboration likelihood model: Implications for the practice of school psychology. J School Psychol.

[36] Ajzen I (1991). The theory of planned behavior. Organiz Behav Hum Decision Proc.

[37] Smit ES, Hoving C, Schelleman-Offermans K, West R, de Vries H (2014). Predictors of successful and unsuccessful quit attempts among smokers motivated to quit. Addict Behav.

[38] Vangeli E, Stapleton J, Smit E, Borland R, West R (2011). Predictors of attempts to stop smoking and their success in adult general population samples: a systematic review. Addiction.

[39] de Vries H, Mudde AN, Dijkstra A, Willemsen MC (1998). Differential beliefs, perceived social influences, and self-efficacy expectations among smokers in various motivational phases. Prev Med.

[40] de Vries H, Mudde A, Leijs I, Charlton A, Vartiainen E, Buijs G, Clemente MP, Storm H, González Navarro A, Nebot M, Prins T, Kremers S (2003). The European Smoking Prevention Framework Approach (EFSA): an example of integral prevention. Health Educ Res.

[41] van den Putte B, Yzer MC, Brunsting S (2005). Social influences on smoking cessation: a comparison of the effect of six social influence variables. Prev Med.

[42] Rains S (2013). The Nature of Psychological Reactance Revisited: A Meta-Analytic Review. Hum Commun Res.

[43] DeLeeuw E (2005). Dropout in longitudinal studies: Strategies to limit the problem. Encycl Stat Behav Sci.

[44] Van Horn PS, Green KE, Martinussen M (2008). Survey Response Rates and Survey Administration in Counseling and Clinical Psychology. Educ Psychol Meas.

