# Supplementary material for: Effectiveness of Message Frame-Tailoring in a Web-Based Smoking Cessation Program: Randomized Controlled Trial
Source: J Med Internet Res. 2020 Apr 3;22(4):e17251. doi: 10.2196/17251 (PMC7165309; doi:10.2196/17251)
Supplement: Multimedia Appendix 1 [file jmir_v22i4e17251_app1.docx]

| **Supplement 1.** *Item wording, and reliability.* |  |  |  |
| --- | --- | --- | --- |
| *Scale, item wording* | *Alpha* | *M* | *SD* |
| *Need for autonomy (HCOS)* | 0.76 | 3.00 | 0.67 |
| Imagine, you want to quit smoking. How likely is it in that case that you decide yourself how to quit? |  |  |  |
| Imagine, you want to quit smoking. How likely is it in that case that you ask an expert (e.g. general practitioner) who tells you how to quit? |  |  |  |
| Imagine, you want to quit smoking. How likely is it in that case that you ask friends and family how to best quit? |  |  |  |
| Setting goals is a good way to quit smoking. How likely is it in that case that you set your own goals? |  |  |  |
| Setting goals is a good way to quit smoking. How likely is it in that case that you ask an expert ( e.g. general practitioner) to set goals for you? |  |  |  |
| Setting goals is a good way to quit smoking. How likely is it in that case that you ask your friends and family to set goals for you? |  |  |  |
| Your health care provider tells you that there are different ways to quit smoking. How likely is it in that case that your first thought is: “What do I think is the best way to quit smoking?” |  |  |  |
| Your health care provider tells you that there are different ways to quit smoking. How likely is it in that case that your first thought is: “What does the expert (e.g. general practitioner) think is the best way for me to quit smoking?” |  |  |  |
| Your health care provider tells you that there are different ways to quit smoking. How likely is it in that case that your first thought is: “What do my friends and family think is the best way for me to quit smoking?” |  |  |  |
| Imagine, you need to find motivation to quit smoking. How likely is it in that case that you find the motivation yourself? |  |  |  |
| Imagine, you need to find motivation to quit smoking. How likely is it in that case that you ask an expert (e.g. general practitioner) to motivate you? |  |  |  |
| Imagine, you need to find motivation to quit smoking. How likely is it in that case that you ask friends and family to motivate you? |  |  |  |
| *Perceived relevance* | 0.87 | 4.44 | 0.08 |
| PAS answers my questions fully ad carefully. |  |  |  |
| PAS allows me to provide input on how I would like to do things. |  |  |  |
| PAS takes into account my emotions in the advice given. |  |  |  |
| I feel that PAS cares about me as a person. |  |  |  |
| *Self-determined motivation (TSRQ)* | 0.92 | 5.34 | 0.21 |
| The reason I would stop smoking is because I feel that I want to take responsibility for my own health. |  |  |  |
| The reason I would stop smoking is because I personally believe stopping smoking is the best thing for my health |  |  |  |
| The reason I would stop smoking is because I personally believe stopping smoking is the best thing for my health |  |  |  |
| The reason I would stop smoking is because I have carefully thought about stopping smoking and believe it is very important for many aspects of my life. |  |  |  |
| The reason I would stop smoking is because stopping smoking is an important choice I really want to make. |  |  |  |
| The reason I would stop smoking is because stopping smoking is consistent with my life goals. |  |  |  |
| *Social norms* | 0.48 | 2.22 | 0.71 |
| My partner would want me to quit smoking. |  |  |  |
| My children would want me to quit smoking. |  |  |  |
| My friends would want me to quit smoking. |  |  |  |
| *Social support* | 0.58 | 3.48 | 1.08 |
| My partner supports to quit smoking. |  |  |  |
| My children support me to quit smoking. |  |  |  |
| My friends support me to quit smoking. |  |  |  |
| *Attitudes pros of smoking cessation* | 0.79 | 3.55 | 0.62 |
| My condition improves when I quit smoking. |  |  |  |
| My health improves when I quit smoking. |  |  |  |
| I feel more attractive when I quit smoking. |  |  |  |
| I feel more satisfied about myself when I quit smoking. |  |  |  |
| I am a better role model when I quit smoking. |  |  |  |
| I am less ashamed of myself. |  |  |  |
| *Attitudes cons of smoking cessation* | 0.75 | 2.36 | 0.51 |
| I will gain weight when I quit smoking. |  |  |  |
| I will be less able to relax when I quit smoking. |  |  |  |
| I will be more bored when I quit smoking |  |  |  |
| I will feel lonelier when I quit smoking |  |  |  |
| I will feel more insecure when I quit smoking. |  |  |  |
| I will feel sadder when I quit smoking. |  |  |  |
| *Self-efficacy* | 0.91 | 3.51 | 0.29 |
| To what extent do you think that you will succeed to refrain from smoking when you take a break? |  |  |  |
| To what extent do you think that you will succeed to refrain from smoking when you feel sad? |  |  |  |
| To what extent do you think that you will succeed to refrain from smoking when you are stressed or tensioned? |  |  |  |
| To what extent do you think that you will succeed to refrain from smoking when you are angry? |  |  |  |
| To what extent do you think that you will succeed to refrain from smoking when someone offers you a cigarette ? |  |  |  |
| To what extent do you think that you will succeed to refrain from smoking when you drink coffee or tea? |  |  |  |
| To what extent do you think that you will succeed to refrain from smoking after a meal? |  |  |  |
| To what extent do you think that you will succeed to refrain from smoking when you see someone else enjoying a cigarette? |  |  |  |
| To what extent do you think that you will succeed to refrain from smoking when you are at a party? |  |  |  |

*Note*. M= mean. SD= standard deviation. Alpha = internal consistency (Cronbach’s alpha). TSRQ= Treatment Self-Regulation Questionnaire. HCOS= Health Causality Orientations Scale; items were rephrased and adapted for the study context of smoking cessation.
