# Supplementary material for: Effectiveness of Message Frame-Tailoring in a Web-Based Smoking Cessation Program: Randomized Controlled Trial
Source: J Med Internet Res. 2020 Apr 3;22(4):e17251. doi: 10.2196/17251 (PMC7165309; doi:10.2196/17251)
Supplement: Multimedia Appendix 2 [file jmir_v22i4e17251_app2.docx]

**Supplement 2**. Correlation matrix of all items (N = 273).

| Variable | Relevance | Motivation | Apro | Acon | SEav | Smoking abstinence^a^ |
| --- | --- | --- | --- | --- | --- | --- |
| Relevance | 1 | .239^**^ | .243^**^ | -.158^**^ | .165^*^ | .153^*^ |
| Motivation |  | 1 | .446^**^ | -.135^*^ | .177^**^ | .048 |
| Apro |  |  | 1 | -.093 | -.111 | -.057 |
| Acon |  |  |  | 1 | -.510^**^ | -.222^**^ |
| SEav |  |  |  |  | 1 | .440^**^ |
| Smoking abstinence^a^ |  |  |  |  |  | 1 |
| *Note.* SEav = Self-efficacy perception about smoking cessation. Acon = Perception of cons of smoking cessation. Apro = Perception of pros of smoking cessation. Motivation = Self-determined motivation to quit smoking. Relevance = Relevance of smoking cessation message. Smoking abstinence = 7-day point-prevalence abstinence of smoking.  ^a^ = Spearman correlation.  * = Correlation is significant at p <0.05. ** = Correlation is significant at p < 0.01. | | | | | | |
