# Supplementary material for: Effectiveness of Message Frame-Tailoring in a Web-Based Smoking Cessation Program: Randomized Controlled Trial
Source: J Med Internet Res. 2020 Apr 3;22(4):e17251. doi: 10.2196/17251 (PMC7165309; doi:10.2196/17251)
Supplement: Multimedia Appendix 3 [file jmir_v22i4e17251_app3.docx]

| **Supplement 3**. Manipulation assessment. | | | | | | | |  |
| --- | --- | --- | --- | --- | --- | --- | --- | --- |
| Item | In this programme, I could choose myself whether I wanted to receive different tips about preparing my quit smoking attempt. | The advice was formulated in a pressuring tone. | In this programme, I could not choose a quit smoking date myself. | The advice told me what I must do instead of what I could do. | In this programme, I received advice based on the responses that I gave to the questions. | The advice was written specifically for me. | The advice was formulated in a way that it would be relevant for everyone | |
| Condition | M (SD) | M (SD) | M (SD) | M (SD) | M (SD) | M (SD) | M (SD) | |
| *Frame-tailoring & content-tailoring* |  |  |  |  |  |  |  | |
| High NFA | 4.69 (0.74)^a^ | 1.73 (1.06)^a^ | 1.42 (0.78)^a^ | 2.24 (1.52)^a^ | 4.35 (1.07)^a^ | 3.06 (1.33)^a^ | 3.39 (1.34)^a^ | |
| Low NFA | 3.64 (1.19)^b^ | 2.50 (1.44)^b^ | 4.06 (1.17)^b^ | 3.17 (1.25)^b^ | 3.94 (1.17)^a^ | 3.14 (1.22)^a^ | 3.53 (1.28)^a^ | |
| *Frame-tailoring & no content-tailoring* |  |  |  |  |  |  |  | |
| High NFA | 4.79 (0.57) ^a^ | 1.71 (1.08)^a^ | 1.57 (1.23)^a^ | 2.00 (1.33)^a^ | 3.96 (1.20)^a^ | 2.68 (1.12)^a^ | 4.14 (1.21)^a^ | |
| Low NFA | 3.07 (1.39)^b^ | 2.80 (1.40)^b^ | 3.77 (1.33)^b^ | 3.23 (1.38)^b^ | 3.23 (.94)^b^ | 2.47 (1.28)^a^ | 3.77 (1.19)^a^ | |
| *No frame-tailoring & no content-tailoring* | 3.84 (1.23)^c^ | 1.75 (.97)^c^ | 1.96 (1.23)^c^ | 2.05 (1.14)^c^ | 3.42 (1.15)^b^ | 2.69 (1.12)^a^ | 4.15 (0.91)^b^ | |
| *No frame-tailoring & content-tailoring* | 3.65 (1.22) ^b^ | 2.11 (1.13)^a^ | 3.71 (1.48)^b^ | 2.72 (1.35)^b^ | 4.19 (1.04)^a^ | 3.07 (1.06)^a^ | 3.43 (1.21)^a^ | |
| *Note*. Means with differing subscripts within rows differ significantly at *p* < 0.05. NFA = Need for autonomy. N = Number of participants. M= Mean. SD = Standard Deviation. | | | | | | | |  |
