# Supplementary material for: Effectiveness of Message Frame-Tailoring in a Web-Based Smoking Cessation Program: Randomized Controlled Trial
Source: J Med Internet Res. 2020 Apr 3;22(4):e17251. doi: 10.2196/17251 (PMC7165309; doi:10.2196/17251)
Supplement: Multimedia Appendix 4 [file jmir_v22i4e17251_app4.docx]

**Supplement 4.** Examples of smoking cessation advice provided in each of the conditions

| Condition | Message frame-tailoring (high & low need for autonomy) | No message frame-tailoring |
| --- | --- | --- |
| Content-tailoring | **Nice** to hear that you wouldn’t feel lonelier when you quit smoking. As other smokers, you are not sure whether you wouldn’t feel sad. Many smokers think that smoking would help them against feelings of loneliness, anger, and sadness. Often, people feel bored and don’t feel like doing anything when being sad. **Below, you can choose whether you would like to receive some tips about how to prevent yourself from negative thoughts and feelings.**  *Choice question*: Do you want to receive these tips?  *Answered “yes”:*  What **could** you do when feeling sad or lonely? **We** **would like** to offer you some tips. You could watch TV, read a book, call (a) friend(s), go outside or repair something. **You could** as well plan something nice, such as going to the cinema or the spa. Maybe it **would help** you to talk about your feelings with someone or write them down. Further, **you may** try not to think about the fact that you miss smoking. **Maybe you** like to distract yourself and think about the nice things, in life, so the negative thoughts and feelings will fade away.  You don’t think that you wouldn’t feel lonelier when you quit smoking. However, you are not sure whether you wouldn’t feel sad. **According to experts**, many smokers think that smoking would help them against feelings of loneliness, anger, and sadness. **Experts say** that many people feel bored and don’t feel like doing anything when being sad. This is why **you must** **watch** TV, read a book, call (a) friend(s), go outside or repair something. **You must look** for distraction when feeling sad or lonely and plan something nice, such as going to the cinema or the spa. Also, **you must share** your feelings with someone or write them down. **Don’t think** about it that you miss smoking. **You have to think** of the nice things in life, then the negative thoughts will fade away. | Nice to hear that you wouldn’t feel lonelier when you quit smoking. However, you are not sure whether you wouldn’t feel sad. Many smokers think that smoking would help them against feelings of loneliness, anger, and sadness. Many people feel bored and don’t feel like doing anything when being sad. You can watch TV, read a book, call (a) friend(s), go outside or repair something. Also, you can look for distraction when feeling sad or lonely and plan something nice, such as going to the cinema or the spa. It’s good to share your feelings with someone or write them down. Don’t think about it that you miss smoking, but think about the nice things in life, then the negative thoughts will fade away. |
| No content-tailoring | Many smokers think that smoking would help them against feelings of loneliness, anger, and sadness. **What could you do when feeling sad or lonely?** Many people feel bored and don’t feel like doing anything when being sad. **You could** watch TV, read a book, call (a) friend(s), go outside or repair something in case you feel sad or lonely. **You could** also look for distraction when feeling sad or lonely and plan something nice, such as going to the cinema or the spa. Also, **you could** share your feelings with someone or write them down when you feel sad. **Maybe you could try** not to think about it that you miss smoking, but about the positive things in life, then negative thoughts and feelings will fade away.  **According to experts,** many smokers think that smoking would help them against feelings of loneliness, anger, and sadness. **Experts say that** many people feel bored and don’t feel like doing anything when being sad. **You must watch** TV, read a book, call (a) friend(s), go outside or repair something In case you feel sad or lonely. **You must** also look for distraction when feeling sad or lonely and plan something nice, such as going to the cinema or the spa. Also, **you must share** your feelings with someone or write them down. Don’t think about it that you miss smoking. You must think about the positive things in life, so negative thoughts will fade away. | Many smokers think that smoking would help them against feelings of loneliness, anger, and sadness. Many people feel bored and don’t feel like doing anything when being sad. In case you feel sad or lonely, you can watch TV, read a book, call (a) friend(s), go outside or repair something. You can also look for distraction when feeling sad or lonely and plan something nice, such as going to the cinema or the spa. Also, it is good to share your feelings with someone or write them down. Don’t think about it that you miss smoking, but about the positive things in life, so negative thoughts and feelings will fade away. |
